# Crystallographic phase identifier of a convolutional self-attention neural network (CPICANN) on powder diffraction patterns

**DOI:** 10.1107/S2052252524005323

**Published:** 2024-06-27

**Authors:** Shouyang Zhang, Bin Cao, Tianhao Su, Yue Wu, Zhenjie Feng, Jie Xiong, Tong-Yi Zhang

**Affiliations:** a Materials Genome Institute Shanghai University Shanghai200444 People’s Republic of China; b Guangzhou Municipal Key Laboratory of Materials Informatics, Sustainable Energy and Environment Thrust, Advanced Materials Thrust, Hong Kong University of Science and Technology (Guangzhou), Guangzhou511400, Guangdong, People’s Republic of China; ESRF, France

**Keywords:** computational modeling, structure prediction, X-ray diffraction, powder diffraction, phase identification, convolutional self-attention, autonomous characterization, neural networks, CPICANN

## Abstract

Spectroscopic data, particularly diffraction data, are essential for materials characterization due to their comprehensive crystallographic information. The current crystallographic phase identification, however, is very time consuming. To address this challenge, we have developed a real-time crystallographic phase identifier based on a convolutional self-attention neural network (CPICANN). Trained on 692 190 simulated powder X-ray diffraction (XRD) patterns from 23 073 distinct inorganic crystallographic information files, CPICANN demonstrates superior phase-identification power. Single-phase identification on simulated XRD patterns yields 98.5 and 87.5% accuracies with and without elemental information, respectively, outperforming *JADE* software (68.2 and 38.7%, respectively). Bi-phase identification on simulated XRD patterns achieves 84.2 and 51.5% accuracies, respectively. In experimental settings, CPICANN achieves an 80% identification accuracy, surpassing *JADE* software (61%). Integration of CPICANN into XRD refinement software will significantly advance the cutting-edge technology in XRD materials characterization.

## Abstract 2

The development of CPICANN, a novel convolutional self-attention neural network, represents a groundbreaking approach in materials informatics. By leveraging the convolutional self-attention mechanism, CPICANN automates and significantly enhances the efficiency of crystal phase identification from whole X-ray powder diffraction patterns, marking a substantial advancement over traditional time-consuming methods.

## 1. Introduction

Efficient materials characterization is pivotal in advancing material design. Extensively big data are generated from various spectroscopic techniques and conventionally necessitate time-consuming expert analysis. This traditional approach significantly slows the pace of materials development and poses challenges for the information feedback in materials fabrication, *in situ* characterization and controllable automated systems, such as artificial intelligent (AI) laboratories (Merchant *et al.*, 2023 ▸; Soldatov *et al.*, 2021 ▸; Peng & Wang, 2023 ▸). In particular, powder diffractometers are widely used to examine the crystallographic structures of polycrystalline solids, which generate powder diffraction patterns. With domain knowledge, analyzing a whole diffraction pattern determines the crystallographic phases and associated microstructures of a tested material. Notably, employing powder diffraction data as a definitive fingerprint for the identification of material crystalline structure is a standard practice in the field. The analysis of a whole diffraction pattern is an extensive search-and-match process across well determined powder diffraction patterns and examined ones. The first step in diffraction-pattern analysis is to find the potential crystal structures and space groups, which provide the peak positions in analyzed diffraction patterns. This is usually carried out manually based on domain knowledge and chemical compositions of diffracted materials. The first step is termed as crystalline-phase identification. It is crucial as it enables the calculation of the static structure factor (Svensson *et al.*, 1980 ▸) based on the diffraction pattern, aiding in the derivation of atom sites during refinement. With existing crystals in databases, the commonly employed method is the search-and-match approach to retrieve potential crystallographic information. Once the basic crystallographic information is obtained, techniques such as pattern decomposition and pattern Rietveld refinements are employed. These methods facilitate the measurement of atom sites, temperatures, grain sizes, residual stress and other detailed structural information, contributing to the comprehensive determination of a crystal structure.

*JADE* (Miettinen *et al.*, 2017 ▸) is a widely used software that provides line- and profile-based approaches for the phase identification. The line-based approach searches and matches only peak locations, recommending comparison of the first 32 lines of a potential crystal. On the other hand, the profile-based approach resembles a visual comparison of *d*–*I* lines (lattice plane distance–intensity) to the experimental trace, assessing similarity between the experimental pattern and potential crystals. The common practice combines the two approaches: first, line matching locates the peak locations to narrow down the search space and then the profile (intensity) matching analyzes the diffraction patterns more deeply. It is a considerable challenge to make the search-and-match automatically and intelligently.

Recently, using AI and machine learning (ML) in the research and development of materials has revolutionized the culture of materials science and engineering (Cao *et al.*, 2024 ▸, 2022 ▸; Xiong *et al.*, 2023 ▸; Xiong & Zhang, 2022 ▸; Takiguchi *et al.*, 2024 ▸; Vollmar, 2022 ▸; Park *et al.*, 2024 ▸; Fuller & Rudden, 2024 ▸; Xu *et al.*, 2023 ▸; Chen *et al.*, 2021 ▸), especially in the crystallographic phase identification area (Pan *et al.*, 2023 ▸; Lee *et al.*, 2021 ▸, 2020 ▸; Szymanski *et al.*, 2023 ▸, 2021 ▸; Maffettone *et al.*, 2021 ▸; Wang *et al.*, 2020 ▸). For example, Lee *et al.* (2020 ▸) employed convolutional neural networks (CNNs) to train on 600 942 simulated spectroscopies, which were derived from mixtures of 38 distinct binary and ternary crystals in Sr–Li–Al–O inorganic compounds (from the Inorganic Compound Structure Database, ICSD). The CNN phase-identification model, after being trained on 600 942 simulated spectroscopies, achieved nearly perfect accuracy across a test dataset of 100 000 simulated instances and 100 experimental patterns of Sr–Li–Al–O compounds. Szymanski *et al.* (2021 ▸) implemented a CNN model to navigate phase identification within the crystallographic space of Li–Mn–Ti–O–F compositions. They compiled 140 distinct stoichiometric phases from the ICSD and complemented them with 115 calculated solid solutions using Vegard’s law, which together formed 255 base phases and associated diffraction patterns. Mixing the 255 patterns with different ratios generated 38 250 patterns. The pre-trained CNN model demonstrated a classification accuracy of 94% across 4200 simulated X-ray diffraction (XRD) patterns and 55% accuracy on 20 experimental profiles in identifying single phases. Maffettone *et al.* (2021 ▸) developed a crystallography companion agent (XCA) that employed an ensemble of CNN models to provide a probabilistic prediction of crystalline phases. This approach adeptly manages the inherent uncertainty in the phase analysis and other uncertainties in the experiment. Notably, the XCA classifications were correct on 56 of the 60 patterns in its application to the phase transitions of BaTiO_3_. Wang *et al.* (2020 ▸) also developed a CNN model on 58 292 simulated patterns derived from 1012 distinguished metal-organic framework crystals, achieving a top-five accuracy of 96.7% in a test dataset of 14 572 instances and 56.7% accuracy for exact identification in 30 experimental XRD patterns. Space groups, rather than crystal types, can be extracted with ML-based analysis from X-ray powder diffraction data (Lee *et al.*, 2023 ▸; Tiong *et al.*, 2020 ▸; Choudhary *et al.*, 2022 ▸), and, in this circumstance, the ML-based analysis covers a much larger number of crystal types with a high prediction accuracy. For instance, Tiong *et al.* (2020 ▸) proposed an advanced multi-stream dense-net framework and achieved an accuracy of 80.12% in identifying the space group within an imbalanced dataset comprising 108 658 crystals from 72 space groups. Salgado *et al.* (2023 ▸) trained a non-pooling CNN model on 171 006 crystallographic information files (CIFs) extracted from the ICSD; the pre-trained model’s performance had a classification accuracy of 67% for the crystal systems and 36% for the space groups of the 2253 inorganic crystals in the Material Project (Jain *et al.*, 2013 ▸) database.

**Figure 1:**
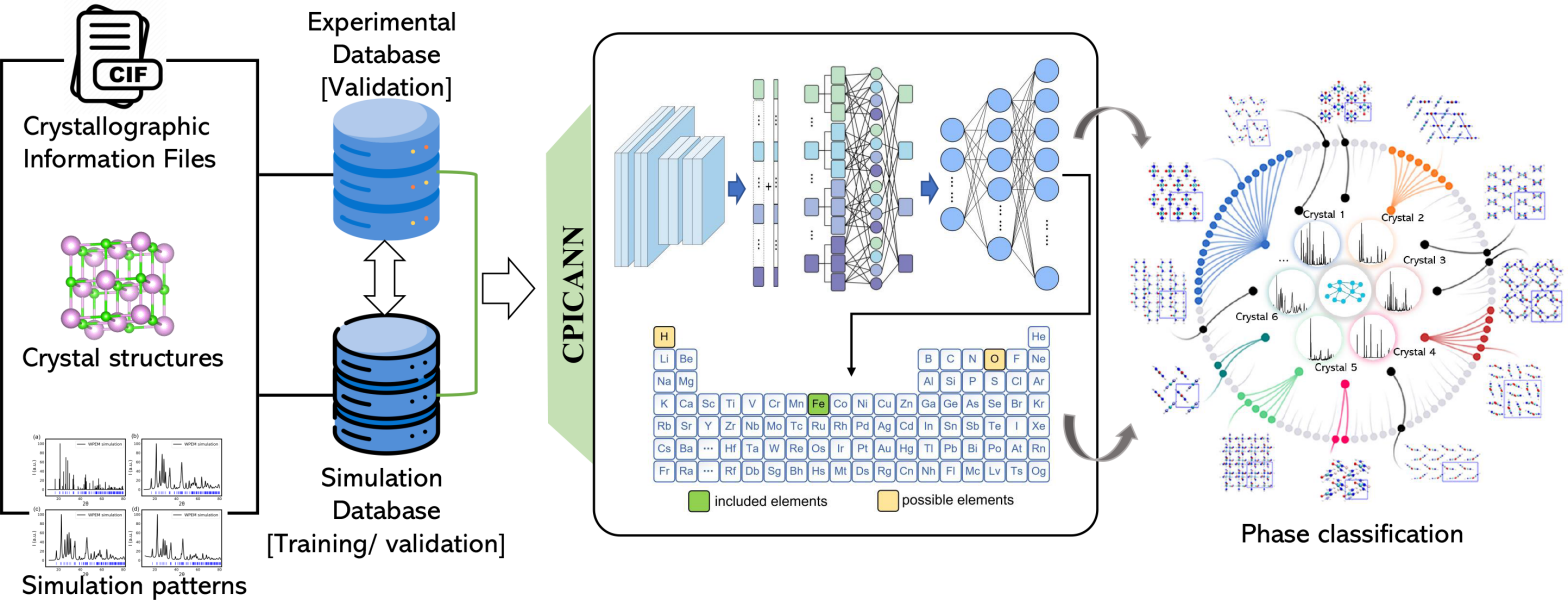
The workflow of CPICANN, where crystal structure is visualized by *VESTA* (Momma & Izumi, 2008 ▸).

The present work develops a crystallographic phase identifier for a convolutional self-attention neural network (CPICANN) to address the practical application and effectiveness challenges of ML-based powder XRD pattern identifications. The CPICANN can recommend the most potential crystal structures in powder XRD patterns for subsequent refinement techniques such as Rietveld refinement (Rietveld, 1967 ▸, 1969 ▸), Le Bail (2005 ▸) and *WPEM* (Cao, 2024 ▸; Qin *et al.*, 2023 ▸). Fig. 1 ▸ shows the workflow of CPICANN, which establishes a basic dataset containing 23 073 basic crystallographic structures of unary, binary and ternary inorganic crystals, retrieved from the Crystallography Open Database (COD) (Gražulis *et al.*, 2009 ▸). With the basic dataset, X-ray powder diffraction patterns of single crystal phases are simulated using the newly developed simulation code. These single-phase patterns are mixed to form X-ray powder diffraction patterns of multiple phases. Subsequently, CPICANN employs convolution and self-attention mechanisms to extract crystal information from the entire powder diffraction patterns without using any elemental information. The pre-trained CPICANN is highly effective in the precise and efficient identification of single phases, while also expediting the swift screening of multi-phase patterns. Proving more effective than conventional line-based approaches, CPICANN is ready for integration into X-ray diffraction refinement software such as *WPEM*, *JADE* and *Fullprof*. Furthermore, the scope of potential applications of CPICANN extends beyond XRD analysis, encompassing various characterization techniques that detect information in the Fourier space, such as electron and neutron diffraction and scattering (Gemmi *et al.*, 2019 ▸; Bacon, 1975 ▸).

## 2. Methods

### 2.1. XRD pattern simulation

A basic crystallographic dataset is built up by selecting 23 073 distinctive CIFs of unary, binary and ternary inorganic crystals from the COD (Gražulis *et al.*, 2009 ▸). The diffraction vectors were determined using the Bragg equation *n*λ = 2*d* sin (θ) to calculate 2θ, where *n* = 1, 2,… is an integer, λ is the wavelength of the X-ray beam, *d* is the crystalline plane spacing and θ = 2θ/2, with 2θ denoting the angle between the incident and diffracted beams. Considering the wide application of Cu anodes, both Cu *K*α_1_ = 1.54056 Å and Cu *K*α_2_ = 1.54433 Å are taken as the wavelengths of the X-ray beam, which makes simulation more practical. In all simulated powder X-ray patterns, 2θ ranges from 10 to 80° with a 0.015° step, and the simulated intensity *I*^*WPEM*^ is expressed by
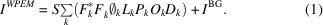
Here, *S* denotes the scale factor, *F*_*k*_ is the structure factor, 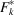 is the complex conjugate of *F*_*k*_, ∅_*k*_ is the profile function, *L*_*k*_ is the Lorentz–polarization factor, *P*_*k*_ represents multiplicity, *O*_*k*_ is the preferred orientation factor, *D*_*k*_ is the Debye factor and *I*^BG^ is the background intensity. A detailed description of all the terms involved in equation (1) is given in Supplementary Note 1 of the supporting information.

Utilizing the *WPEM* simulation module, we implement six distinct data-augmentation approaches to enhance the diversity of simulated patterns. (1) Peak broadening is effectively modeled by adjusting the grain size from 2 to 50 nm. Instrumental broadening manifests in experimental patterns due to convolution with the crystal peak. Typically, during refinement, observed peaks are separated into Voigt and instrumental components. However, quantifying the instrumental component proves challenging as it varies with the diffraction angle. Therefore, in our simulations, we opt for a smaller grain size than realistic to accurately replicate the degree of experimental broadening (Maniammal *et al.*, 2017 ▸). (2) An orientation effect arises from the uneven distribution of small grains in the incident beam, leading to an uneven distribution of reciprocal sites across the reciprocal spheres of the powder specimen. To simulate the impact of random orientation, we adopt the approach used by Szymanski *et al.* (2021 ▸), adjusting the peak intensity within a 40% range. (3) A thermal vibration effect is simulated by allowing atoms to shift from their average positions within a range of 0.05–0.5 Å. (4) Internal stress is simulated by randomly adjusting the lattice constant by up to 20% for the structure factor. (5) Instrument zero shift is simulated by randomly translating 2θ within −3–3°. (6) Specimen contamination is simulated by randomly blending the diffraction signal of another crystal within a mixture ratio of 5–30%. To assemble a comprehensive simulated pattern, all of the above six data-augmentation techniques are applied across 30 iterations within the designated parameters, resulting in 30 diffraction patterns for each individual crystal. Thus, a total of 692 190 simulated single-phase powder XRD patterns were generated.

Various levels of background and noise were introduced into a total of 692 190 simulated patterns to assess their effects. Dataset 1 was devoid of background but included Gaussian noise with a standard deviation (σ) of 0.25. Dataset 2 featured a 3% background ratio along with Gaussian noise (σ = 0.25). Datasets 3 and 4 consisted of Gaussian noise with standard deviations of σ = 1 and σ = 3, respectively, without any background. Table S2 in Supplementary Note 2 of the supporting information shows that CPICANN’s accuracy in single-phase identification without elemental information was 86.98% for the 3% background mixture, 86.35% for the noise mixture of σ = 1 and 84.30% for the noise mixture of σ = 3. Evidently, high levels of background or noise adversely affect CPICANN’s performance. All datasets and corresponding pre-trained models are publicly accessible. Specific background-stripping algorithms and smoothing techniques could alleviate these challenges during real-world analysis. Nevertheless, while striving for high-throughput autonomous characterization, some degree of accuracy may need to be compromised. However, subsequent discussions in this work will focus on dataset 1, which presents minimal noise interference, to concentrate on the phase-identification challenge.

**Figure 2:**
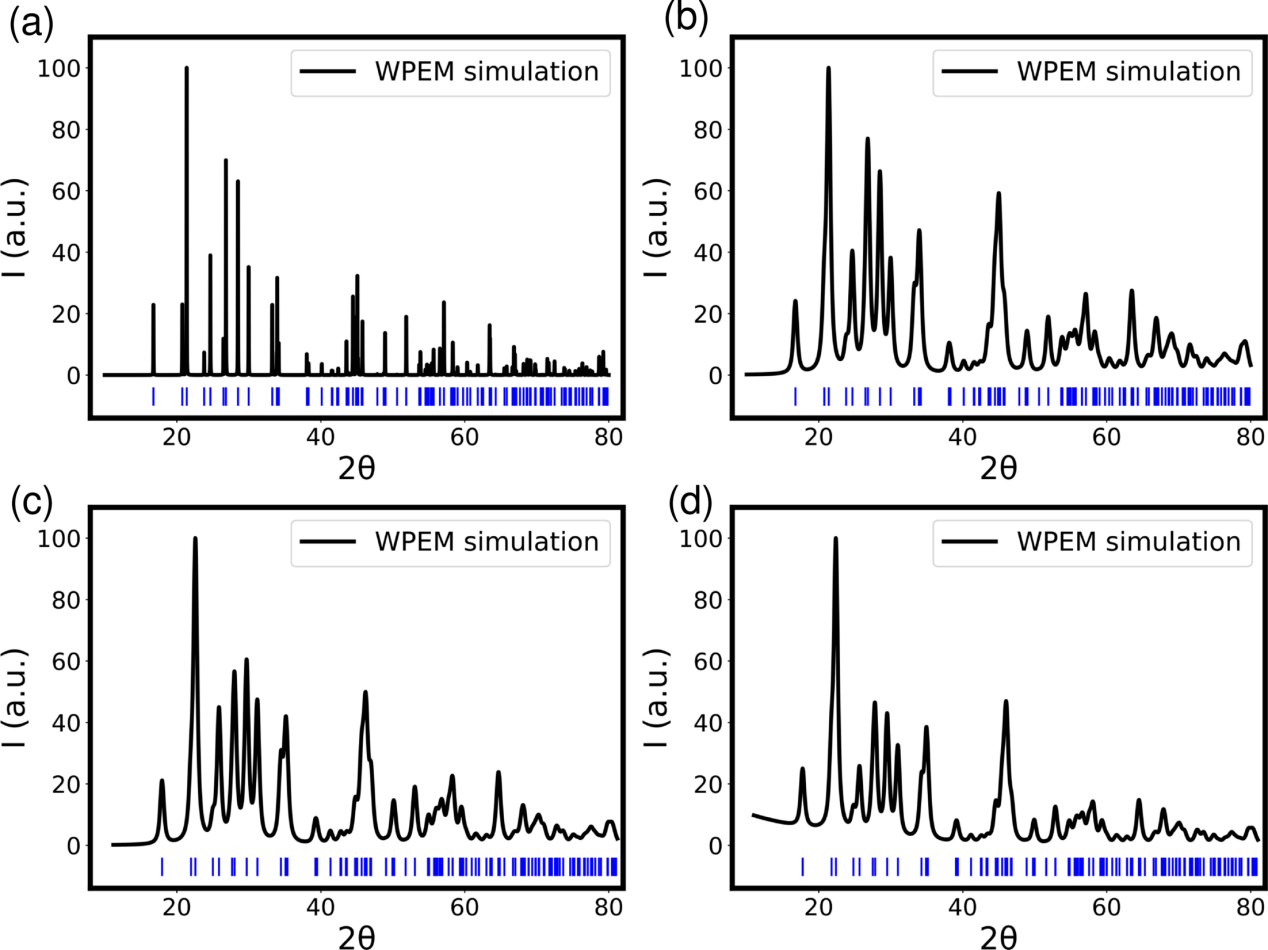
(*a*)–(*d*) Simulated X-ray powder diffraction patterns of PbSO_4_ crystal under a Cu anode. (*a*) An ideal crystal; (*b*) an average grain size of 3 nm; (*c*) an orientation factor of 0.3, a thermal-vibration derivation of 0.2 and a zero shift of 1.2°; and (*d*) with background intensity.

Figs. 2 ▸(*a*)–2 ▸(*c*) demonstrate the sequential integration of the six augmentation types. Fig. 2 ▸(*a*) displays a simulated X-ray powder diffraction pattern of an ideal PbSO_4_ crystal, covering the 2θ range of 10–80° and encompassing a total of 108 diffraction peaks. Notably, with an average grain size set at 3 nm, many peaks overlap due to the peak-broadening effect, as seen in Fig. 2 ▸(*b*). Fig. 2 ▸(*c*) introduces factors such as the orientation factor, lattice alteration and a 1.2° zero shift, leading to deviations in peak intensities and positions from theoretical expectations. Fig. 2 ▸(*d*) depicts the pattern with added background, revealing a subtle rise in the low-angle pattern.

In addition to the single-phase patterns, binary-phase XRD patterns were generated by leveraging the 692 190 simulated single-phase patterns. This was achieved by blending two patterns with the formula *py*_1_ + (1 − *p*)*y*_2_, where *y*_1_ and *y*_2_ represent selected single-phase patterns and *p* denotes the mixing ratio, ranging from 0.2 to 0.8. This blending approach assumes that no reaction occurs between the phases. A pre-process on powder XRD patterns is carried out by selecting 4500 points from each XRD pattern within the 2θ range from 10 to 80° with a step size of 0.015° so that the corresponding intensities are expressed as a 4500 × 1-dimensional vector, which is hence standardized. The intensity for each specific diffraction angle is assigned based on the experimental patterns provided. In this study, we identify the nearest diffraction angle on the experimental pattern and allocate the corresponding intensity to the 4500-dimensional input vector. In cases where two angles are equally close to the matched angle, we select the one with the higher intensity.

### 2.2. Convolutional self-attention neuron network

A series of ablation experiments were conducted to determine the architecture of the CPICANN framework. The input vector is processed through a dedicated convolutional block designed to mitigate the potential impact of subtle background and noise (He *et al.*, 2016 ▸). Our findings indicate that the convolutional block enhances robustness, particularly in scenarios characterized by subtle noise. Additionally, the inclusion of a self-attention mechanism enables the incorporation of both local and global contextual information from XRD patterns, significantly improving the performance of CPICANN over conventional CNN models that lack self-attention blocks. Without the self-attention module, the CNN module has a prediction accuracy of 79.86% on the validation set, which is lower than the accuracy of 87.50% of CPICANN on the validation set (see Supplementary Note 4 for details). Based on the prediction accuracy on the validation set, the self-attention module is optimized within the following ranges: self-attention layers of 4, 6 or 8; embedding dimensions of 128, 256 or 384; and head numbers of 4, 6 or 8. The results are listed in Table S5 with the notations of ED for embedding dimensions, HN for head number and SL for the number of self-attention layers. The ablation study yields the optimal configuration of CPICANN: ED = 128, HN = 8 and SL = 6. For comprehensive details of the ablation experiments, please refer to Supplementary Note 4.

**Figure 3:**
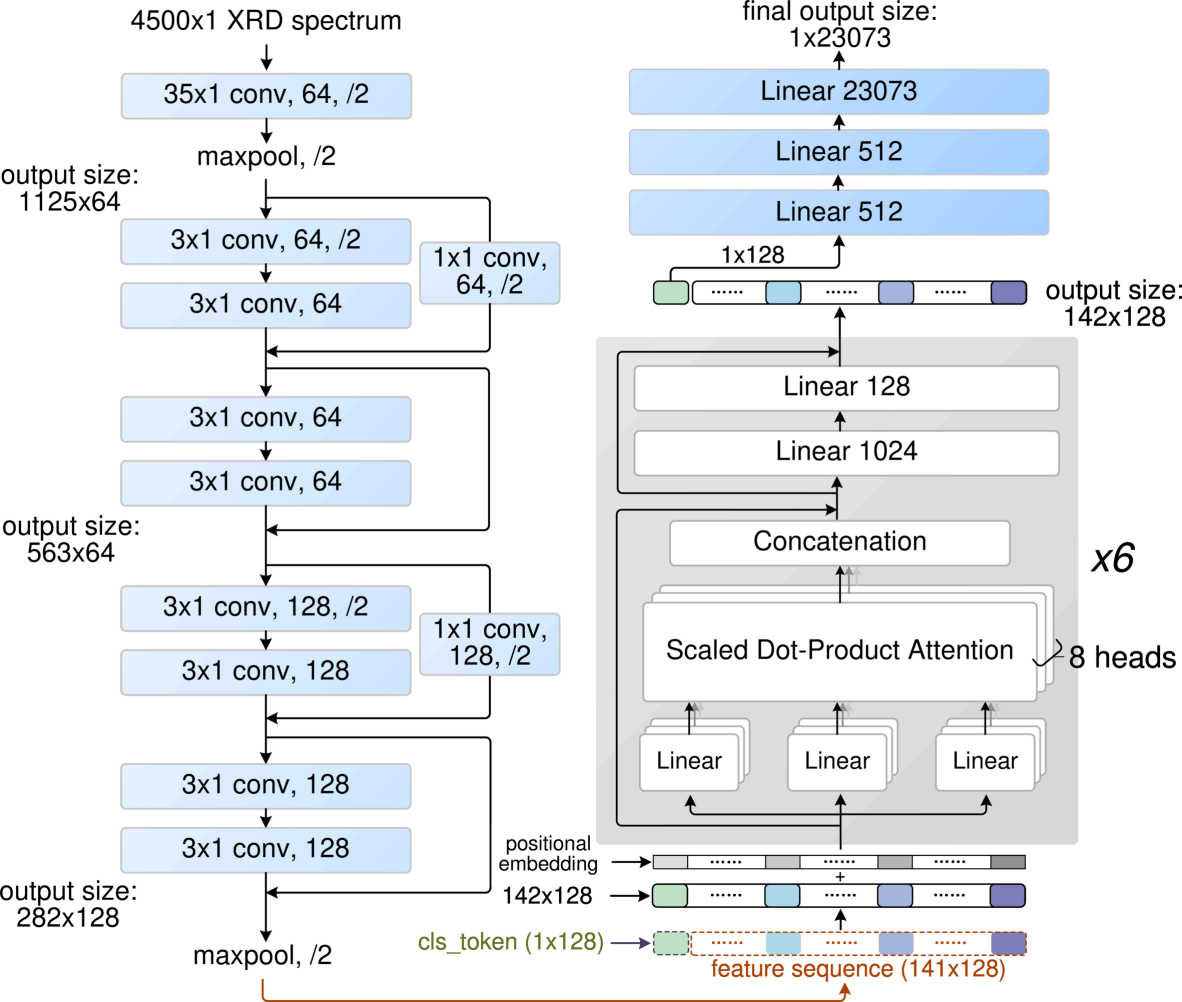
The architecture of CPICANN. In each of the one-dimensional convolution layers, *n* × 1 conv., *m* and /2 denote the kernel size *n*, the channel number *m* and a stride of 2, respectively. In the max-pooling layers, /2 also indicates a stride of 2. Residual connection is indicated by solid lines. The convoluted information is fed into six eight-head self-attention blocks, which scores the input XRD pattern against the 23 073 single-phase patterns.

The finalized architecture of CPICANN comprises a convolution block, six self-attention blocks and multilayer perceptron (MLP) layers, as depicted in Fig. 3 ▸. The convolution block consists of nine convolutional layers that generate 128 channels of 141-dimensional vectors from the 4500 × 1-dimensional XRD pattern input. These vectors are treated as a sequence of 141 tokens, with each token having the embedding vector of 128 elements provided by the 128 channels. By introducing a classification token (cls_token) as the start token, the 142 × 128 matrix with its positional embeddings (Vaswani *et al.*, 2017 ▸) is input to six eight-head self-attention blocks (Dosovitskiy *et al.*, 2020 ▸). The 128-dimensional embeddings are uniformly split into the eight heads, with each head handling 16-dimensional embeddings. The outputs of each head are then concatenated, resulting in a feature sequence with the same dimension of 142 × 128. This sequence is sent to two MLP layers, which linearly map the sequence to 142 × 1024 and then back to 142 × 128. Finally, the embeddings of the cls_token(1 × 128) are fed through three MLP layers to obtain the category probability output. In summary, CPICANN houses a total of 14 385 505 trainable parameters.

For single-phase identification, the 692 190 simulated XRD patterns are randomly separated into two sets, 553 752 for training and 138 438 for testing. After training, the parameters in the convolutional block of CPICANN are fixed, and the 14 146 337 trainable parameters are retrained on 1.6 billion patterns for bi-phase identification, where the testing patterns are 400 million. The focal cross entropy (Lin *et al.*, 2020 ▸) and cross entropy loss functions (Galstyan & Cohen, 2007 ▸) (see Supplementary Note 3) are adopted for single-phase and bi-phase identifications, respectively, providing probabilistic rather than determinate predictions over possible crystals. This allows CPICANN to recommend top-scoring phases for user consideration and use with a parameter-free element filter.

CPICANN incorporates elemental information by using an element filter like that used in *JADE*. During the inference process with elemental information, the filter is applied on the model output and categorizes all elements in the periodic table into three groups: *A* ‘included elements’, *B* ‘possible elements’ and *C* ‘excluded elements’, *viz*. all elements are *A* ∪ *B* ∪ *C*. In the XRD pattern identification, the included elements must appear simultaneously with variation in their individual concentrations, the possible elements can possibly appear individually and the excluded elements cannot appear at all. For example, if Fe is the included element and S and O are possible elements, the XRD patterns for crystals Fe, FeS, Fe_2_O_3_, Fe_3_O_4_, Fe_2_(SO_4_)_3_, *etc*. form a set *S*, much smaller than the whole set, and an analyzed XRD pattern will be matched with those in set *S*. But, the XRD pattern for crystal FeCl_2_, for example, does not belong to the set *S* because Cl is one of the excluded elements.

## 3. Results and discussion

### 3.1. Single-phase identification

**Figure 4:**
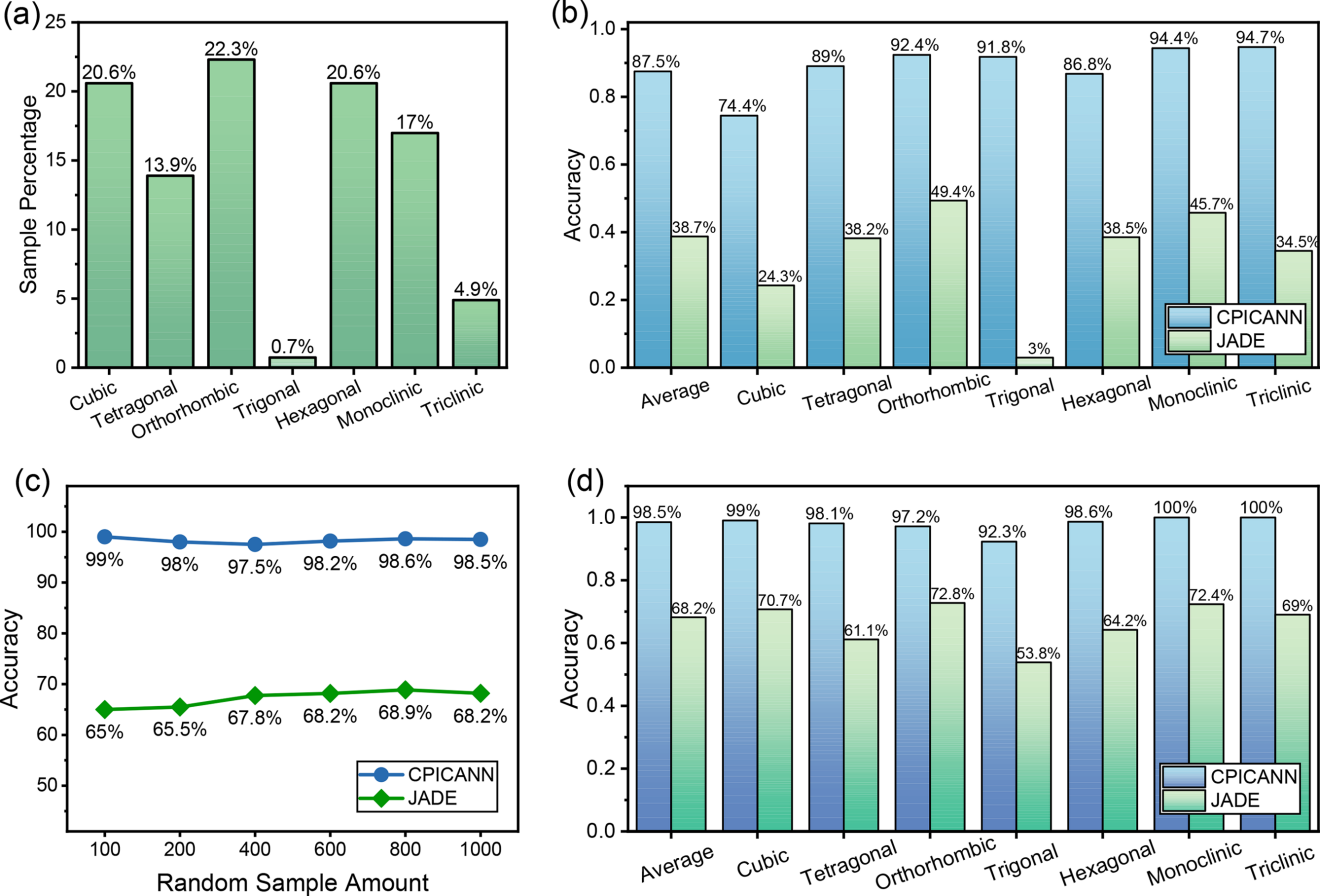
(*a*) The data distribution in the seven crystal systems for both the training and testing datasets. (*b*) The performances of CPICANN and Task-Macro in *JADE* on the single-phase identification in each of the seven crystal systems. (*c*) The performance accuracy versus random sample amounts of CPICANN and *JADE* on the single-phase identification with elemental information, where the accuracy is averaged over the seven crystal systems. (*d*) The performances of CPICANN and *JADE* on the single-phase identification over 1000 random XRD patterns in each of the seven crystal systems.

The performance of CPICANN is benchmarked against the line-based search and match (LBSM) algorithm from *JADE* (*JADE Pro* 8.9) in scenarios both with and without elemental information. Primarily, the fundamental assessment of CPICANN focuses on the scenario devoid of elemental information. The Task-Macro module in *JADE* (Miettinen *et al.*, 2017 ▸), which is the widely used code for element-free scenarios, is used here for comparison. Fig. 4 ▸(*b*) shows the performances of CPICANN and the Task-Macro module in *JADE* on the single-phase identification across the seven crystal systems. Fig. 4 ▸(*a*) illustrates the data percentages for these crystal systems in both the training and testing datasets, which maintain identical proportions derived from the utilized CIFs. The orthorhombic crystal system has the largest number in the training and testing datasets, and both CPICANN and Task-Macro exhibit their own greatest identification accuracy for this crystal system: 92.4% for CPICANN and 49.4% for Task-Macro. As expected, CPICANN significantly outperforms the Task-Macro module, nearly doubling its identification accuracy. It is surprising to see that CPICANN also exhibits excellent performance in the trigonal crystal system, although it has the smallest data in both training and testing sets, with an accuracy of 91.8%. In contrast, the Task-Macro module performs extremely poorly in the trigonal crystal system, with only a 3% identification accuracy. The most outstanding performances of CPICANN are in the triclinic and monoclinic crystal systems, with identification accuracies of 94.7 and 94.4%, respectively. In comparison, Task-Macro reaches only 34.5 and 45.7% in these systems. It appears that CPICANN performs better in crystal systems with less symmetry, as illustrated in Fig. 4 ▸(*b*). This trend suggests that lower symmetric space groups have more features and are therefore easily captured by CPICANN. Overall, the average identification accuracy is 87.5% for CPICANN across all 138 438 testing simulated patterns, significantly surpassing the 38.7% achieved by Task-Macro in *JADE* on the same testing XRD patterns.

In practice, the elemental information is usually required when using *JADE* for LBSM identification. Therefore, the effectiveness of CPICANN was further assessed under conditions where the elemental information is available. Since manual operation should be carried out on individual XRD patterns in *JADE* for LBSM identification with elemental information, ten small datasets, each comprising 100 XRD patterns, were selected without replacement from the testing dataset for this evaluation. These selections maintain the same ratios of the seven crystal systems shown in Fig. 4 ▸(*a*). Fig. 4 ▸(*c*) shows the identification accuracy, averaged over the seven crystal systems, versus the sample amount for both CPICANN and *JADE*. As expected, providing elemental information enhances the identification accuracy for both methods. Although there are still some fluctuations in the accuracy as the sample number increases, these fluctuations are extremely small when the sample number exceeds 600, indicating statistical reliability. The CPICANN and LBSM results on 1000 samples are detailed in Fig. 4 ▸(*d*). With the inclusion of elemental information, CPICANN achieves exceptional performance, especially in the triclinic and monoclinic crystal systems, reaching 100% identification accuracy. Even in the cubic crystal system, the provision of elemental information increases the identification accuracy from 74.4% to 99%. Similarly, the performance of the LBSM identification of *JADE* improves significantly with the addition of elemental information, most notably in the trigonal crystal system, where the identification accuracy increases from 3% to 53.8%. Overall, across the seven crystal systems, CPICANN performs outstandingly with an accuracy of 98.5%, and *JADE* also achieves a notable performance, reaching an average accuracy of 68.2%. CPICANN significantly outperformed *JADE* in the single-phase identification across both scenarios, whether elemental information was provided or not.

### 3.2. Bi-phase identification

**Figure 5:**
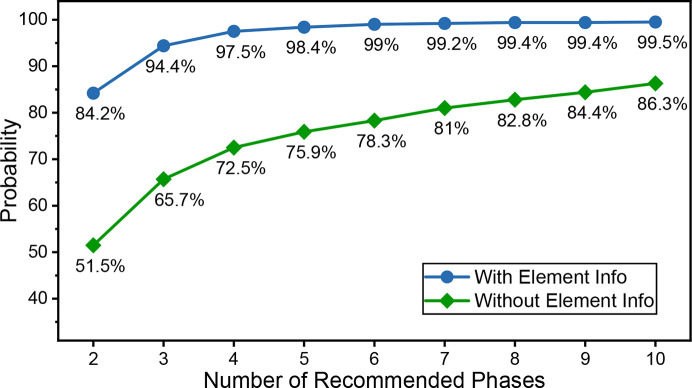
The probability of the right two phases in the recommended phases as a function of the number of recommended phases with and without elemental information.

With the cross entropy loss function, the trained CPICANN model recommends the top probabilities of two–ten phases, which include the correct two phases in an examined bi-phase XRD pattern, with and without elemental information. Based on 20 000 testing bi-phase simulated XRD patterns, Fig. 5 ▸ shows the probability of the right two phases in the recommended phases as a function of the number of recommended phases, with and without elemental information. If only two phases are recommended, CPICANN achieves an identification accuracy of 51.1% without any elemental information and 84.2% with it. When three phases are recommended, the probabilities of finding the right two phases among the three recommendations are 94.4% with and 65.7% without elemental information. As expected, the probability of correctly identifying the correct two phases increases with the number of phases recommended, as depicted in Fig. 5 ▸. Furthermore, it might be sufficient for CPICANN to recommend four phases when elemental information is provided, as the probability already reaches 97.5%. Conversely, without elemental information, more recommended phases are required to achieve a high probability of success. Obviously, recommending more phases requires greater downstream refinement, indicating the significant role of elemental information in the phase identification for powder XRD patterns.

### 3.3. Experimental practice

**Figure 6:**
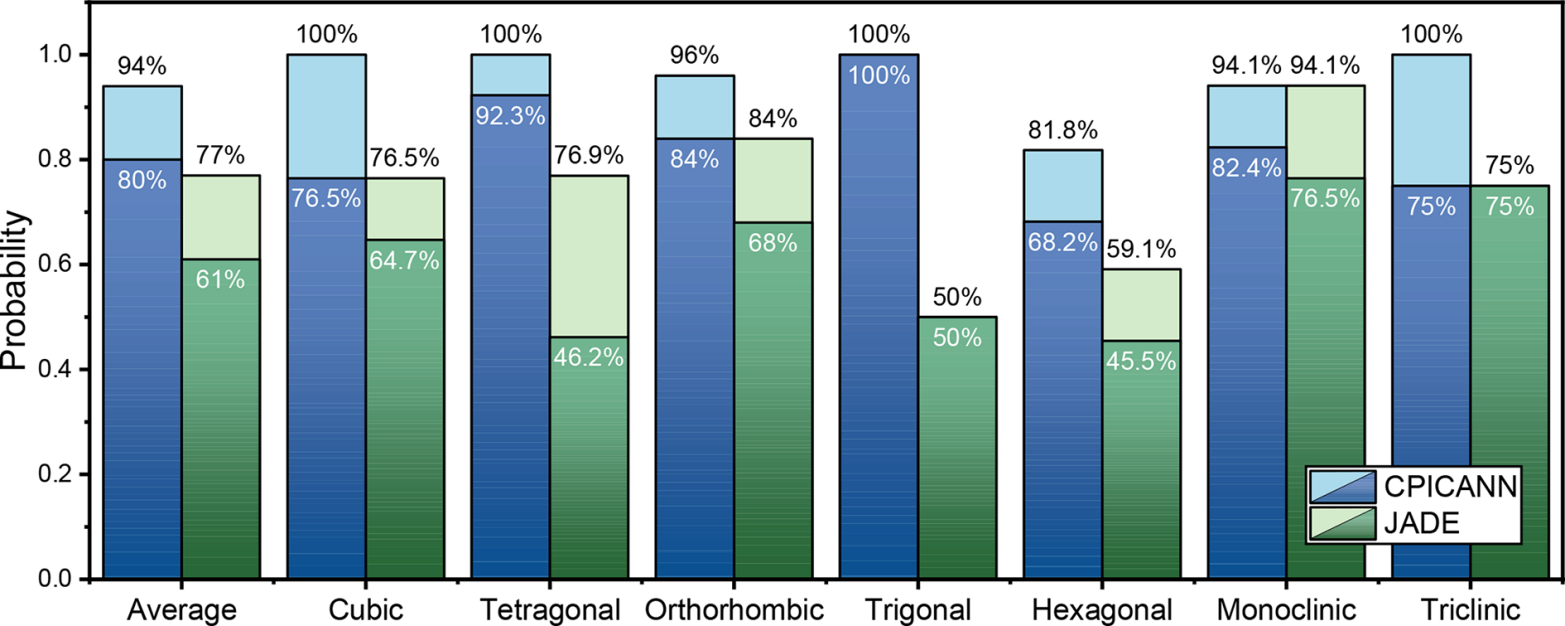
The probability of the right phase in the phases recommended by CPICANN and *JADE*, where the dark and light colors mark recommended one and three phases, respectively.

The phase-identification capability of CPICANN, when equipped with elemental information, was further assessed with 100 single-phase experimental powder XRD patterns, comprising ten from our own laboratory and 90 from the RRUFF database (Lafuente *et al.*, 2015 ▸). This test on experimental data is crucial to evaluating the applicability and effectiveness of CPICANN in real-world experiments. Detailed information about these 100 single-phase experimental powder XRD patterns can be found in Tables S6 and S7, and is accessible via the *XRED* platform (https://www.github.com/WPEM/XRED). Fig. 6 ▸ illustrates the performance of CPICANN on these 100 experimental XRD patterns across the seven crystal systems, with a comparative analysis of the performance of *JADE*. When only one phase is recommended by CPICANN and *JADE*, their performance is assessed by identification accuracy. CPICANN shows exceptional performance in the trigonal crystal system, achieving 100% identification accuracy, whereas *JADE* achieves only 50% identification accuracy in the same crystal system. Averaged across the seven crystal systems of the 100 XRD patterns, the identification accuracies are 61% for *JADE* and 80% for CPICANN, clearly exhibiting the merit of CPICANN. If more phases are recommended, the performance is measured by the probability of correctly identifying the phase within the recommended set. As depicted in Fig. 5 ▸, an increase in the number of recommended phases leads to a higher probability of correct identification. However, selecting the correct phase from a larger set of recommendations becomes more challenging. As a showcase, Fig. 6 ▸ shows the identification probabilities when three phases are recommended. In this circumstance, CPICANN significantly outperforms *JADE*, particularly in the cubic, tetragonal and triclinic crystal systems, where the identification probability reaches 100%.

## 4. Concluding remarks

The present work develops a novel network, CPICANN, for crystal phase identification on whole X-ray powder diffraction patterns, utilizing a convolutional self-attention mechanism. CPICANN can automate and integrate the XRD patterns into a unified attention-matching strategy. The performance and effectiveness of CPICANN are extremely powerful, as shown here by the single-phase and bi-phase identifications with and without elemental information on simulated XRD patterns, and the single-phase identification on experimental XRD patterns. Elemental information is initially provided manually in the conventional identification approach. In contrast, CPICANN employs elemental information afterwards, applying it only to those highly potential crystal phases selected based on their attention probability scores from the examined whole XRD pattern. This merit of CPICANN minimizes potential errors in the elemental information provided.

The success of CPICANN in phase identification represents a significant advancement in materials informatics, providing a more efficient and accurate method for automatic phase identification and rapid screening in complex material crystal structures. In future work, we will integrate CPICANN with the XRD refinement software *WPEM* to develop an AI-driven XRD analyzer.

## 5. Code availability

The model described in the present work was implemented in Python. Source codes are available at https://www.github.com/WPEM/CPICANN.

## 6. Related literature

The following references are only cited in the supporting information for this article: Armstrong (1967 ▸), Caglioti *et al.* (1958 ▸), Gerward (1993 ▸) and Nguyen *et al.* (2014 ▸).

## Supplementary Material

Supporting information. DOI: 10.1107/S2052252524005323/fc5077sup1.pdf

## Data Availability

All data studied in this article are available at https://www.github.com/WPEM/CPICANN.
