# Supplementary material for: Crystallographic phase identifier of a convolutional self-attention neural network (CPICANN) on powder diffraction patterns
Source: IUCrJ. 2024 Jun 27;11(Pt 4):634–42. doi: 10.1107/S2052252524005323 (PMC11220882; doi:10.1107/S2052252524005323)
Supplement: Supplementary file 1 [file m-11-00634-sup1.pdf]

IUCrJ

Volume 11 (2024)

Supporting information for article:

Crystallographic phase identifier of a convolutional self-attention neural network (CPICANN) on powder diffraction patterns

Shouyang Zhang, Bin Cao, Tianhao Su, Yue Wu, Zhenjie Feng, Jie Xiong and Tong-Yi Zhang

Content :

Supplementary Note 1: XRD pattern simulation

Supplementary Note 2: Performance of CPICANN trained on data with noise and background

Supplementary Note 3: The configuration and training procedures of CPICANN

Supplementary Note 4: Ablation study

Table S1-S7

Figure S1

Reference

Supplementary Note 1.

XRD pattern simulation.

The simulated intensity at each 2θ, I^{WPEM}, is expressed by

$$I^{WPEM} = S\sum_{k}\left(F_k^{*}F_k\emptyset_k L_k P_k O_k D_k\right) + I^{BG}. \tag{1}$$

In Eq. 1, S denotes the scale factor, F_k the structure factor, F_k^* the complex conjugate of F_k, $\emptyset_k$ the peak profile function, L_k the angle factor, P_k the multiplicity, O_k the preferred orientation factor, D_k the Debye-Waller factor, and I^{BG} the background intensity. The structure factor (F_k) is computed by

$$F_k = \sum_{j=1}^{N} f_j e^{2\pi i \mathbf{G}^*\cdot\mathbf{R}}, \tag{2}$$

where f_j is the form factor (Gerward, 1993), R the lattice coordinate of atom j, and n the total number of atoms in a lattice cell. The peak profile function ($\emptyset_k$) is a Voigt function (Armstrong, 1967), viz.,

$$\emptyset_k = \frac{1}{\sigma\pi\sqrt{2\pi}}\int\left(\left[\frac{\gamma}{(2\theta-t)^2+\gamma^2}\right]\cdot\exp\left(-\frac{(2\theta-t)^2}{2\sigma^2}\right)\right)dt. \tag{3}$$

The peak broadening is represented by the Full Width at Half Maximum (FWHM, Γ), where $2\gamma = 2\sqrt{2ln2}\sigma = \Gamma$ (Caglioti *et al.*, 1958). The angle factor L_k is calculated by

$$L_k = \frac{1+\cos^2 2\theta}{\sin^2\theta\cos\theta}, \tag{4}$$

where 2θ denotes the diffraction angles. The multiplicity P_k is determined by counting the number of diffraction vectors present within an Ewald diffraction sphere. The Debye-Waller factor D_k is calculated by

$$D_k = e^{-2M}, \tag{5}$$

$$M = \frac{6h^2T}{mk\Theta^2}\left(\emptyset\left(\frac{\Theta}{T}\right)+\frac{\Theta}{4T}\right)\frac{\sin^2\theta}{\lambda}, \tag{6}$$

where h is Planck's constant, m is atom mass, k is Boltzmann constant, Θ is the Debye temperature, T is absolute temperature, and $\emptyset\left(\frac{\Theta}{T}\right)$ is the Debye function.

Supplementary Note 2.

Performance of CPICANN trained on data with noise and background

The simulation of the background is achieved through polynomial fitting. Ten spline points are randomly selected within the range defined by the table below. Subsequently, a polynomial function of power 6 is employed to fit and extrapolate all background values across diffraction angles. The background intensity is then integrated into the original pattern at a ratio of 3% to 97%, resulting in the derivation of the background-contained diffraction pattern.

Table S1. *Background spline points ranges*

Points	2θ	Intensity	Points	2θ	Intensity
1	10°	0-10	6	40°-50°	2-5
2	15°-30°	10-15	7	50°-60°	2-5
3	40°	15-20	8	60°-70°	2-5
4	30°-40°	15-20	9	70°-80°	2-5
5	35°	5-10	10	80°	0-5

Table S2. *Performance of CPICANN trained on data with noise and background.*

No.	Remark	Accuracy on validation set (%)
**dataset 1**	**0% background ratio and Gaussian noise (σ=0.25)**	**87.50**
dataset 2	3% background ratio and Gaussian noise (σ=0.25)	86.98
dataset 3	0% background ratio and Gaussian noise (σ=1)	86.35
dataset 4	0% background ratio and Gaussian noise (σ=3)	84.30

Three Gaussian noise levels $N(0, \sigma)$, ($\sigma = 0.25$), ($\sigma = 1$) and ($\sigma = 3$), and a 3% background with Gaussian noise level $\sigma = 0.25$, are added on the 692,190 simulated patterns. The table above shows that the accuracy of CPICANN in single phase identification without element information is 86.98% for 3% background ratio, 87.50% for ($\sigma = 0.25$), 86.35% for ($\sigma = 1$) and 84.30% for ($\sigma = 3$).

Supplementary Note 3.

The configuration and training procedures of CPICANN.

Figure 3 (main paper) shows the configuration of CPICANN. In each of the one-dimensional convolution layers, n×1, conv. m, /2 denote the kernel size n, channel number m and a stride of 2, respectively. In the max-pooling layers, /2 indicates also a stride of 2. Residual connection is indicated by solid lines. The working flow is described here in detail. The i-th XRD pattern is encoded with a normalized 4500×1 dimensional vector $\mathbf{X}^1_{i,4500}\,(i = 1, \ldots, N)$ as input data with N patterns, where the superscript denotes a single channel and 4500 means that 4500 points of 2θ are uniformly taken from a studied XRD whole pattern, which values vary from pattern to pattern. The 4500 points behave like 4500 pixels in one-dimensional images.The vector $\mathbf{X}^1_{i,4500}$ is input into a 1-dimensional convolution layer with 64 channels and a kernel size of 35 with zero padding on the front and end 17 pixels and a stride of 2. The first convolution layer delivers an $\mathbf{X}^{64}_{i,2250}$ tensor. Subsequently, this tensor is fed into a max-pooling layer with kernel size of 3, zero padding on the front and end 1 pixels, and a stride of 2, which results in an $\mathbf{X}^{64}_{i,1125}$ tensor. The zero padding on the front and end 1 pixels is taken as granted without any description hereafter and any marks in Figure 3. The $\mathbf{X}^{64}_{i,1125}$ tensor is simultaneously fed into two convolution layers, one of which has two sublayers. The two sublayers have 64 channels and a kernel size of 3 in each, but strides of 2 and 1 respectively. The single layer is 1-dimensional convolution layer with 64 channels, a kernel size of 1, and a stride of 2. The outputs of the two layers are pointwise added and this operation might be called two-path connection. Then the information $\mathbf{X}^{64}_{i,563}$ goes through another two sublayers with the same structure and operation as described above. This time, residual connection is applied, which means that

$$Res(\mathbf{u}) = \mathbf{u} + f(\mathbf{u}), \tag{7}$$

where $f(\mathbf{u})$ is an operation on the input $\mathbf{u}$. The residual connection partially maintains the original information after taking the new operation $f(\mathbf{u})$. The residual connection is similar to the skip connection and also shown by the solid lines in Figure 3. These layers with two-path connection and residual connection are called a convolution component. After that, another convolution component is designed to information $\mathbf{X}_{i,563}^{64}$ to $\mathbf{X}_{i,141}^{128}$, as illustrated in Figure 3. Finally, the convolution block delivers an $\mathbf{X}_{i,141}^{128}$ tensor as its output. Clearly, the original 1D encoding is converted into the 128D encoding with the 128 channels and the sequence of 4500 points is converted the sequence with 141 tokens. A classification (cls) token, as the start token with the same embedding size, added to the 141 convolutional tokens and here the input $\mathbf{X}_{i,142}^{128}$ is fed into the multi-head self-attention block.

First, positional embeddings (Vaswani *et al.*, 2023) are conducted on $\mathbf{X} \in \mathbb{R}^{142\times 128}$, where whose elements on the ith row and the $(2j)^{th}$ column are expressed by the sine functions and on the ith row and the $(2j+1)^{th}$ column by the cosine functions, viz.,

$$PE_{pos,2j} = \sin\left(\frac{pos}{10000^{2j/128}}\right), \tag{8}$$

$$PE_{pos,2j+1} = \cos\left(\frac{pos}{10000^{2j/128}}\right), \tag{9}$$

where $j = 1, \cdots, 128/2$ and pos denotes the location. The location sequence $E_{i,142}^{128}$ is pointwise added to the convolutional sequence $\mathbf{X}_{i,142}^{128}$,

$$h_{i,142}^{128} = \mathbf{X}_{i,142}^{128} + E_{i,142}^{128}, \tag{10}$$

and subsequently is fed into the six 8-head self-attention blocks. The 128-dimensional embeddings are uniformly divided into 8 folds with each having 16-dimensional embeddings $h_{i,142}^{16}$ for each head.

For each head, an input tensor $h_{i,142}^{16}$ is passed through three separate fully connected neural networks to produce key, query, and value matrices:

$$\mathbf{Q}_{i,142}^{16} = FCL_Q\left(h_{i,142}^{16}\right), \mathbf{K}_{i,142}^{16} = FCL_K\left(h_{i,142}^{16}\right), \mathbf{V}_{i,142}^{16} = FCL_V\left(h_{i,142}^{16}\right). \tag{11}$$

The scaled dot-product attention based on $\mathbf{Q}_{i,142}^{16}, \mathbf{K}_{i,142}^{16}, \mathbf{V}_{i,142}^{16}$ is then calculated as:

$$Attention\left(\mathbf{Q}_{i,142}^{16}, \mathbf{K}_{i,142}^{16}, \mathbf{V}_{i,142}^{16}\right) = softmax\left(\frac{\mathbf{Q}_{i,142}^{16}\mathbf{K}_{i,142}^{16}{}^{T}}{\sqrt{d_k}}\right)\mathbf{V}_{i,142}^{16}, \tag{12}$$

where $d_k = 16$. This attention operation results in a 142×16 dimensional feature sequence as $h_{i,142}^{16}$ from a single scaled dot-product attention head. The outputs of the 8 heads are then concatenated to produce a tensor $\mathbf{X}_{i,142}^{128}$. It is subsequently passed through a multilayer perceptron layer (MLP) comprising two linear layers, resulting in a tensor $\mathbf{X}_{i,142}^{128}$. The six multi-head self-attention blocks are connected sequentially, yielding an output of $\mathbf{X}_{i,142}^{128}$. Finally, the last token of $\mathbf{X}_{i,142}^{128}$ is extracted as $\mathbf{X}_{i}^{128}$, and fed into an MLP composed of three linear layers, reshaping the token dimensions to 1×512, 1×512, and 1×23073 respectively.

During the inference process that provides element information, element filter is applied on the model output. Our approach is similar to the Chemistry Filter in JADE, all elements are categorized into three sets of elements "included elements", "possible elements" and "exclude elements" by user, denoted as A, B and C respectively. All crystals that can be formed by the elements in sets A and B formed a much narrower set of crystal phases, S. A match score of -10^9 is assigned to all phases that are not contained within S. For example, given "Fe" as set A, "S, O" as set B, then all elements other than "Fe", "S" and "O" are to be excluded. Crystals such as "Fe", "FeS", "Fe_2O_3", "$Fe_2(SO_4)_3$", etc. belong to set S, but crystals such as "$FeCl_2$", "Fe_3C", "$FeCO_3$", "$CaCO_3$", etc. don't belong to set S since they contain at least one of the exclude elements.

The output masked by element filter is then passed through a SoftMax activation function, resulting in a vector $\hat{\mathbf{Y}}_{i}^{23073}$ corresponding to 23073 categories probability. If element information is not available, the element filter will be skipped, and the model output will be directly fed into SoftMax.

We chose Focal Loss (Lin *et al.*, 2018) to facilitate single-phase model training. For model output $\hat{\mathbf{Y}}_i^{23073}$, the focal loss is calculated by

$$\mathcal{L}_i = -\left(1 - p_i\right)^{\gamma} \log\left(p_i\right), \tag{13}$$

$$p_i = \frac{\exp\left(\hat{Y}_{i,GT}^{23073}\right)}{\sum_{j=1}^{C} \exp\left(\hat{Y}_{i,j}^{23073}\right)}, \tag{14}$$

where GT is the target for sample i, $C = 23073$ denotes the category is the studied system. $\gamma \geq 0$ is the preset focusing parameter. We set γ to the default value of 2. By adding a modulating factor $(1 - p_i)^{\gamma}$ to the cross-entropy loss, samples that are difficult to classify receive more weight, improving model accuracy.

Multi-phase model is fine-tuned based on pretrained single-phase model. The convolutional layers are frozen since the feature of XRD pattern haven't significantly changed from single-phase to multi-phase. The parameters of self-attention module and MLP layers were reset to avoid single-phase model bias. We employed a soft-label cross entropy loss function (Nguyen *et al.*, 2014; Galstyan & Cohen, 2007), designed to output probabilities for different CIFs, denoted as:

$$\mathcal{L}_i = -\sum_{c=1}^{C} \log \frac{\exp\left(\hat{Y}_{i,c}^{23073}\right)}{\sum_{j=1}^{C} \exp\left(\hat{Y}_{i,j}^{23073}\right)} Y_{i,c}^{23073}, \tag{15}$$

where $\hat{\mathbf{Y}}_i^{23073}$ is the model output, $\mathbf{Y}_{i,c}^{23073}$ is the classification label for category c, $C = 23073$ denotes the category is the studied system.

We designed an average-label scheme for multi-phase classification label setting. Given 2 XRD patterns y_1 and y_2, the one-hot classification label can be expressed as $[0, \ldots, 1, \ldots, 0, \ldots, 0]$ and $[0, \ldots, 0, \ldots, 1, \ldots, 0]$. The blended pattern y is given by $qy_1 + (1 - q)\, y_2$, where $q \in [0.2,\ 0.8]$. For any blend ratio q, the classification label of y for multi-phase classification is always set as $[0, \ldots, 0.4, \ldots, 0.4, \ldots, 0]$. This scheme increases the weight of the pattern that account for a relatively small proportion of the blended XRD pattern, facilitate the model to predict both original patterns.

A 2-stage dynamic learning rate scheme was implemented during both single-phase and multi-phase training. In the first stage, which is the warm-up stage, the learning rate gradually increases to the preset basic learning rate in 20 epochs. The learning rate during warm-up is given by:

$$l_w = l_{base} \frac{epoch_c}{epoch_w}, \tag{16}$$

where $epoch_c$ is the current epoch and $epoch_w$ is the epochs in warm-up stage, which is 20. In the second stage, learning rate slowly decrease by:

$$l_w = 0.5 l_{base} \left(1 + \cos \frac{epoch_c - epoch_w}{epoch_t otal - epoch_w} \right). \tag{17}$$

The training hyperparameters are listed as:

Table S3. *Hyperparameter Setting*

	Single-phase model training	Multi-phase model training
base learning rate	8e-5	8e-4
batch size	128	512
optimizer	AdamW with weight decay of 1e-4	
total training epochs	200	2000
warmup epochs	20	50
loss function	Focal Loss (Lin *et al.*, 2018)	soft-label cross entropy

Supplementary Note 4.

Ablation study.

Table S4. *Ablation study on self-attention mechanism and CNN.*

No.	Model	Trainable Parameters	Accuracy on validation set (%)
**1**	**CPICANN**	**14,385,505**	**87.50**
2	CNN	12,406,127	79.86
3	w/o CNN	14,168,993	84.71

We conducted ablation experiment on the accuracy improvement introduced by self-attention mechanism and CNN. The table above shows that without self-attention module, the CNN model obtained 79.86% accuracy, reduced by 7.64% compared to the best model, and the self-attention module obtained 84.71% accuracy without CNN.

The self-attention module is removed from CPICANN and Figure S1 shows the structure of the CNN network, where the output linear layers remain unchanged and the 141×128 output from the CNN module is mapped a 1×128 vector via an MLP. The CNN model has the prediction accuracy 79.86% on the validation set, which is lower than the accuracy 87.50% of CPICANN on the validation set.

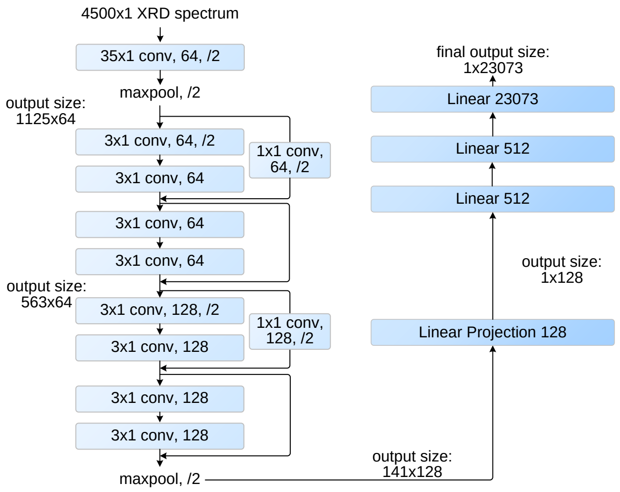

Figure. S1. The structure of the CNN.

Table S5. *Ablation study on self-attention configuration.*

No.	Model	Trainable Parameters	Accuracy on validation set (%)
1	ED: 128, HN: 8, SL: 4	13,725,793	86.16
**2**	**ED: 128, HN: 8, SL: 6**	**14,385,505**	**87.50**
3	ED: 128, HN: 8, SL: 8	15,045,217	86.94
4	ED: 256, HN: 8, SL: 6	17,243,873	85.51
5	ED: 384, HN: 8, SL: 6	20,872,161	86.14
6	ED: 128, HN: 4, SL: 6	14,385,505	86.43
7	ED: 384, HN: 6, SL: 6	20,872,161	85.78

Supplementary Tables.

Table S6. *Experimental powder XRD patterns from our laboratory.*

No.	Chemical Formula	Space group	Crystal system
1	Mn2O3	I2_13	Cubic
2	MnS	Fm-3m	Cubic
3	PbSe	Fm-3m	Cubic
4	Zr	P6_3/mmc	Hexagonal
5	CdS	P6_3mc	Hexagonal
6	NiO2H2	P-3m1	Hexagonal
7	Al2O3	R-3c	Hexagonal
8	β-Sn	I4_1/amd	Tetragonal
9	WO3	P2_1/c	Monoclinic
10	PbSO4	Pnma	Orthorhombic

Table S7: **Experimental powder XRD patterns from RRUFF.**

**No.**	**RRUFF ID**	**Ideal Chemistry**	**Crystal system**
1	R040012	Ca(SO4)	Orthorhombic
2	R040035	Zn(CO3)	Hexagonal
3	R040036	Ba(SO4)	Orthorhombic
4	R040077	Mg(OH)2	Hexagonal
5	R040106	Ce(PO4)	Monoclinic
6	R040131	CaSiO3	Triclinic
7	R040133	Mn(CO3)	Hexagonal
8	R040172	Ca(WO4)	Tetragonal
9	R050044	Pb(CrO4)	Monoclinic
10	R050045	CaF2	Cubic
11	R050072	HgS	Hexagonal
12	R050140	ZnS	Cubic
13	R050143	Ag5SbS4	Orthorhombic
14	R050166	KCl	Cubic
15	R050262	Fe(CO3)	Hexagonal
16	R050287	Na2NaAlF6	Monoclinic
17	R050298	Pb(CO3)	Orthorhombic
18	R050300	Fe2O3	Hexagonal
19	R050322	Cu5FeS4	Cubic
20	R050327	Cu3BiS3	Orthorhombic
21	R050335	Ba(SO4)	Orthorhombic
22	R050355	Ca(MoO4)	Tetragonal
23	R050365	Pb9As4S15	Hexagonal
24	R050390	Mn2+Mn3+6O8(SiO4)	Tetragonal
25	R050391	VO2	Orthorhombic
26	R050403	Cu1+Fe3+O2	rhombohedral
27	R050426	Pb2+2Pb4+O4	Tetragonal

Continued on next page

Table S7 – continued from previous page

No.	RRUFF ID	Ideal Chemistry	Crystal system
28	R050442	Cu3AsS4	Orthorhombic
29	R050449	Al2SiO5	Orthorhombic
30	R050476	Sr(CO3)	Orthorhombic
31	R050480	AgSbS2	Monoclinic
32	R050550	HgSe	Cubic
33	R050561	Al2SiO5	Orthorhombic
34	R050626	Al2SiO5	Orthorhombic
35	R050676	Mg(CO3)	Hexagonal
36	R060067	PbSiO3	Monoclinic
37	R060121	NiAs	Hexagonal
38	R060134	AuTe2	Monoclinic
39	R060197	Al2SiO5	Orthorhombic
40	R060440	Fe7S8	Monoclinic
41	R060546	AlO(OH)	Orthorhombic
42	R060639	CuAgSe	Tetragonal
43	R060655	PbCl2	Orthorhombic
44	R060791	CuFeSe2	Tetragonal
45	R060855	Na2Si2O5	Monoclinic
46	R060925	Ce(PO4)	Monoclinic
47	R060928	NiSb	Hexagonal
48	R060939	PbTe	Cubic
49	R061062	ZnMn3+2O4	Tetragonal
50	R061074	Ni3S2	rhombohedral
51	R061077	Bi2S3	Orthorhombic
52	R070097	Mg2Si2O6	Orthorhombic
53	R070164	AuTe2	Monoclinic
54	R070177	FeSb2S4	Orthorhombic
55	R070211	Mn2+TiO3	Hexagonal
56	R070235	HgO	Orthorhombic
57	R070279	Au	Cubic
58	R070307	CuBiS2	Orthorhombic
59	R070343	NiAsS	Cubic
60	R070368	Y(AsO4)	Tetragonal
61	R070479	MgTiO3	Hexagonal
62	R070530	Ca2(SiO4)	Monoclinic
63	R070534	NaCl	Cubic
64	R070578	Ag2S	Monoclinic
65	R070592	FeAs2	Orthorhombic
66	R070616	WS2	Hexagonal
67	R070730	Cu2S	Monoclinic
68	R080064	CaTa2O6	Orthorhombic
69	R080091	UTi2O6	Monoclinic
70	R080121	NiO	Cubic

Continued on next page

Table S7 – continued from previous page

No.	RRUFF ID	Ideal Chemistry	Crystal system
71	R080122	NiS	Hexagonal
72	R090010	Nb(BO4)	Tetragonal
73	R090012	Fe2+2Ge4+O4	Cubic
74	R090018	(UO2)(CO3)	Orthorhombic
75	R100131	Al2O3	Hexagonal
76	R100133	MgAl2O4	Cubic
77	R100159	Al2OSiO4	Triclinic
78	R100171	ZrO2	Monoclinic
79	R110002	CoAsS	Monoclinic
80	R110021	Cu3SbS4	Tetragonal
81	R110058	CeF3	Hexagonal
82	R110178	CaTiO3	Orthorhombic
83	R120013	TiO2	Tetragonal
84	R120016	CaSiO3	Triclinic
85	R130003	ThO2	Cubic
86	R130024	CuCo2S4	Cubic
87	R130062	AgAsS2	Hexagonal
88	R130093	Al(OH)3	Triclinic
89	R150130	Ca(WO4)	Tetragonal
90	R150142	FeTiO3	Hexagonal

References

Armstrong, B. (1967). *Journal of Quantitative Spectroscopy and Radiative Transfer*, **7**(1), 61–88. https://www.sciencedirect.com/science/article/pii/002240736790057X

Caglioti, G., Paoletti, A. & Ricci, F. (1958). *Nuclear Instruments*, **3**(4), 223–228. https://www.sciencedirect.com/science/article/pii/0369643X5890029X

Galstyan, A. & Cohen, P. R. (2007). In *International Conference on Inductive Logic Programming*, pp. 98–111. Springer.

Gerward, L. (1993). *Radiation Physics and Chemistry*, **41**(4), 783–789. https://www.sciencedirect.com/science/article/pii/0969806X9390326P

Lin, T.-Y., Goyal, P., Girshick, R., He, K. & Dollár, P. (2018). Focal loss for dense object detection.

Nguyen, Q., Valizadegan, H. & Hauskrecht, M. (2014). *Journal of the American Medical Informatics Association*, **21**(3), 501–508.

Vaswani, A., Shazeer, N., Parmar, N., Uszkoreit, J., Jones, L., Gomez, A. N., Kaiser, L. & Polosukhin, I. (2023). Attention is all you need.
